# In Silico Insights towards the Identification of NLRP3 Druggable Hot Spots

**DOI:** 10.3390/ijms20204974

**Published:** 2019-10-09

**Authors:** Nedra Mekni, Maria De Rosa, Chiara Cipollina, Maria Rita Gulotta, Giada De Simone, Jessica Lombino, Alessandro Padova, Ugo Perricone

**Affiliations:** 1 Drug Discovery Unit, Fondazione Ri.MED, 90133 Palermo, Italy; nmekni@fondazionerimed.com (N.M.); ccipollina@fondazionerimed.com (C.C.); mrgulotta@fondazionerimed.com (M.R.G.); gdesimone@fondazionerimed.com (G.D.S.); jlombino@fondazionerimed.com (J.L.); apadova@fondazionerimed.com (A.P.); 2 Istituto per la Ricerca e l’Innovazione Biomedica (IRIB)-Consiglio Nazionale delle Ricerche, 90146 Palermo, Italy; 3 Dipartimento STEBICEF, Università degli Studi di Palermo, 90100 Palermo, Italy

**Keywords:** NLRP3 modulation, MCC950, NACHT domain, walker B, homology modeling, docking, induced-fit docking, molecular dynamics

## Abstract

NLRP3 (NOD-like receptor family, pyrin domain-containing protein 3) activation has been linked to several chronic pathologies, including atherosclerosis, type-II diabetes, fibrosis, rheumatoid arthritis, and Alzheimer’s disease. Therefore, NLRP3 represents an appealing target for the development of innovative therapeutic approaches. A few companies are currently working on the discovery of selective modulators of NLRP3 inflammasome. Unfortunately, limited structural data are available for this target. To date, MCC950 represents one of the most promising noncovalent NLRP3 inhibitors. Recently, a possible region for the binding of MCC950 to the NLRP3 protein was described but no details were disclosed regarding the key interactions. In this communication, we present an in silico multiple approach as an insight useful for the design of novel NLRP3 inhibitors. In detail, combining different computational techniques, we propose consensus-retrieved protein residues that seem to be essential for the binding process and for the stabilization of the protein–ligand complex.

## 1. Introduction

NLRP3 (NOD-like receptor family, pyrin domain-containing protein 3) inflammasome is a cytosolic complex that coordinates innate immunity responses by sensing a wide range of damage- and pathogen-associated molecular patterns (DAMPs and PAMPs). Upon activation, NLRP3 assembles with the adaptor ASC and pro-caspase-1 promoting caspase-1-dependent cleavage of pro-IL-1β, pro-IL-18, and Gasdermin D, leading to cytokine release, pore formation, and eventually pyroptosis [1].

Increased activation of NLRP3 has been related to several chronic pathologies, including neurodegenerative diseases, atherosclerosis, type-II diabetes, fibrosis, and rheumatoid arthritis. Preclinical evidences support that inhibition of NLRP3 can revert the pathological phenotype in several disease models, with minor impairment of host immune defenses. Therefore, NLRP3 represents an appealing pharmacological target. Recently, many research groups have been focusing on the development of selective modulators of the NLRP3 inflammasome, without interfering with the protective activity of other types of inflammasome.

However, the lack of structural information regarding the oligo-protein and the unclear molecular mechanism of known inhibitors hinders the task of designing novel NLRP3 modulators.

NLRP3 structurally includes an N-terminal pyrin domain, a central adenosine triphosphatase (ATPase) domain, known as NACHT, and a C-terminal leucine-rich repeat (LRR) domain. MCC950, a sulfonylurea-structure based compound, has been reported as a potent inhibitor of NLRP3 [2]. Recent studies [3,4,5] showed that MCC950 inhibits NLRP3 in a noncovalent way, and seems to bind in the region proximal to the Walker B motif (296–315), within the NACHT domain, thus affecting the protein activity. However, its binding mode still remains unclear, and the crucial residues involved in the host–guest recognition mechanism are unknown. In the present work, a homology model of the protein was developed and used in a computational workflow to unveil putative protein hot spots involved in the stabilization of the protein–ligand complex with well-known inhibitors (used as a probe), in order to better understand their molecular mechanism.

## 2. Results and Discussion

### 2.1. Homology Modeling

The crystal structure of *Mus Musculus* NLRC4 (NLR family CARD domain-containing protein 4, PDB id: 4KXF) cocrystallized with ADP bound to the nucleotide binding site was chosen as template for our model [6,7]. The homology model was prepared using Phyre2 web services from the human NLRP3 FASTA sequence by www.uniprot.org. The homology model reported 100% confidence, indicating a high accuracy, despite an Identity Sequence of 22% [8]. The model was then carried out further in our computational investigation.

### 2.2. Binding Site Detection

As previously mentioned, the binding site of MCC950 on NLRP3 has not been clearly defined. However, very recently, it has been unveiled that MCC950 binds the NACHT domain, in a region most likely proximal to the Walker B [3,4,5]. In order to gain further insights into the protein hot spots involved in the binding process of MCC950, all the potential druggable sites of the NLRP3 protein were explored. In particular, a binding site is defined as a protein cavity or surface on which a substrate (protein, hormone, drug, enzyme) interacts forming the protein–ligand complex. To this purpose, we envisioned three different approaches using docking-based, grid-based, and geometry-based algorithms, respectively. FTMap [9], SiteMap [10], and fpocket [11,12] were selected for the analysis of the protein structure druggability. FTMap is a computational mapping server that uses 16 small molecules differing in size, shape, and polarity as probes to map the protein. FTMap clusters each probe type per protein region, thus identifying potential protein binding hot spots. On our model, FTMap clustered six out of 16 available probes within the Walker B. SiteMap is a grid-based binding site detector integrated in the Schrödinger small molecule suite. Based on hydrophobicity and hydrogen bond-donor and -acceptor properties, SiteMap reported five putative binding sites, out of which two were included within the Walker B region. The third approach used was fpocket, a geometry-based pocket detector based on Voronoi tessellation of atoms in proteins, built by the qhull package (qvoroinoi) and on alpha sphere. Specifically, between the residues Phe257 and Ala344, an interesting pocket was found, including 34 amino acids (F257, I259, V264, S265, L266, V267, T268, Q296, R270, S271, L272, L275, C279, C280, P281, D2842, P283, N284, P285, P286, I287, K289, I290, V291, F299, M301, D302, L307, L318, S333, L335, L340, P342, and A344). The resulting Pocket score of 33.8884 and a Drug score of 0.8586 were in agreement with fpocket parameters [12], translating into a good probability to be an actual binding site. An in-depth analysis of this sequence confirmed that this potential binding site falls within the Walker B region. These evidences taken together underline the importance of the region near the Walker B motif as potential active site for the binding of small molecules to NLRP3. Gratifyingly, our in silico evidences seem to be aligned with recently published results [3,4,5].

### 2.3. Molecular Docking and Induced-Fit Docking

**Figure 1:**
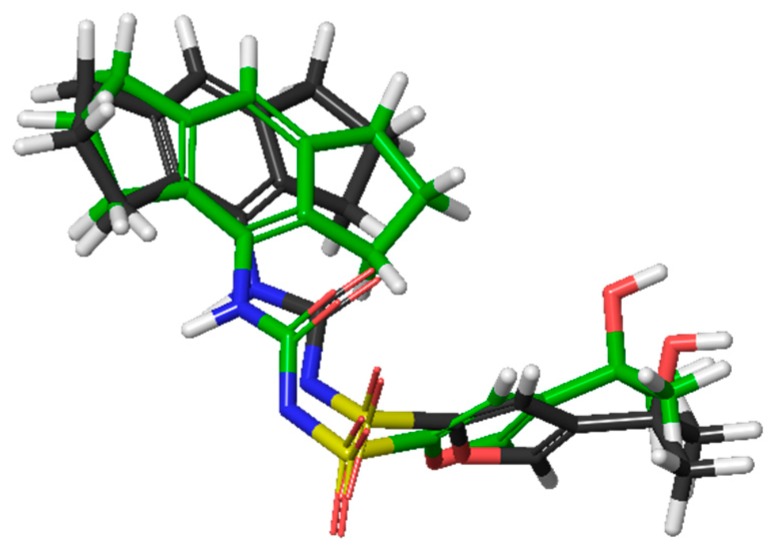
In grey, the MCC950 optimized in DFT, and in green, the same molecule optimized with EpiK (oxygen atoms are displayed in red, hydrogens in white, sulfurs in yellow and nitrogen in blue).

In order to elucidate the protein druggable hot spots, a docking study and molecular dynamics simulations (MD) were performed using MCC950 inhibitor as probe. We initially assessed the quality of the LigPrep tool (Epik) comparing the obtained results for MCC950 with a DFT ligand optimization outcomes (Jaguar) [13]. Based on the results and considering the optimal overlay (RMSD = 1.10 Å), as shown in Figure 1, we decided to continue our study using an Epik conformer for further steps.

Extra precision (XP) docking was run using MCC950 as probe on our NLRP3 homology model to catch putative hot spots on the Walker B, using the Glide (Schrödinger LLC) docking tool [14,15,16]. We performed two docking studies of MCC950, with or without ATP bound, in order to compare our results to the experimental data previously published [3]. When ATP was absent, a docking score of −6.044 Kcal/mol was obtained, a result less favorable than compared to the docking score of −8.850 Kcal/mol, obtained when ATP was bound to NLRP3. Based on our results showing a stronger binding energy when ATP was bound to the protein, and perfectly aligned to evidences in literature, we decided to go further in our study using the model bound to ATP.

It was decided to extend the docking grid mapping including the residues between the 257 and 344 amino acid positions. Starting from Walker B [4,5] we decided to focus our docking evaluation on a wider region based on fpocket results. The larger binding region considered led us to deepen a possible binding site over the Walker B, therefore including the crossing region between the Walker A (ATPase site) and Walker B. For the docking process, the van der Waals radii (*vdW)* scaling factor was set as 0.8 and the partial charge cut off was 0.15, both during the docking grid generation and the docking process. This allowed to have more tolerance for atomic clashes during the binding pose searching phase. The docking was performed with OPLS3e Force Fields with nonimposed constrains [14,15,16]. Binding affinity was evaluated according to docking scores. Moreover, the protein–ligand complex was analyzed considering noncovalent bonds, such as hydrogen bonds, salt bridge, π interactions, and eventually bad contacts or clashes. XP precision docking afforded a score of −8.164 Kcal/mol.

**Figure 2:**
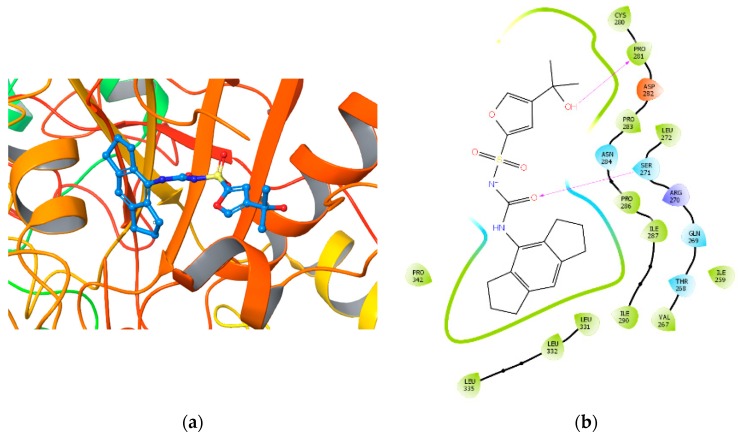
(**a**) The MCC950 binding pose from the extra precision (XP) docking experiment; (**b**) ligand interaction diagram of the MCC950 binding pose. Purple arrows refer to H-bond interactions, green lines around molecule underline non-polar regions whereas the blue ones point polar residues.

The best five binding poses obtained by XP docking converged. In particular, hydrogen bonds were found between Ser271 and the carbonyl urea oxygen of the inhibitor MCC950 while the tertiary alcohol showed interaction with the Pro281 and Asn284 of the protein. The complex was stabilized by hydrophobic interactions of Cys280, Leu332, and Leu335. Figure 2a depicts the best XP docking pose result, whereas Figure 2b shows putative interactions between the ligand MCC950 and NLRP3.

To validate the XP retrieved pose, a second docking study was performed, rebuilding the docking grid on a reduced sequence centered only on the Walker B region. On this grid, we performed XP docking experiments.

**Figure 3:**
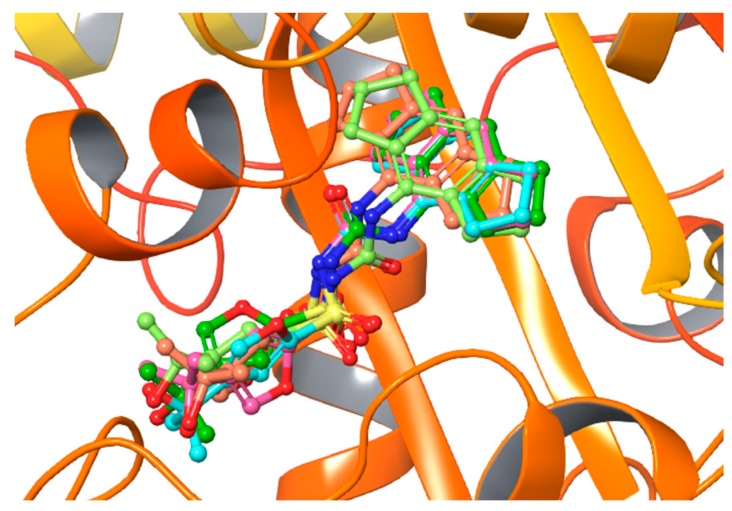
The five best poses of MCC950 from the XP docking experiment on the centered Walker B region.

The best XP docking score was −8.850 Kcal/mol and all docking poses converged. The best docking results are shown in Figure 3.

**Figure 4:**
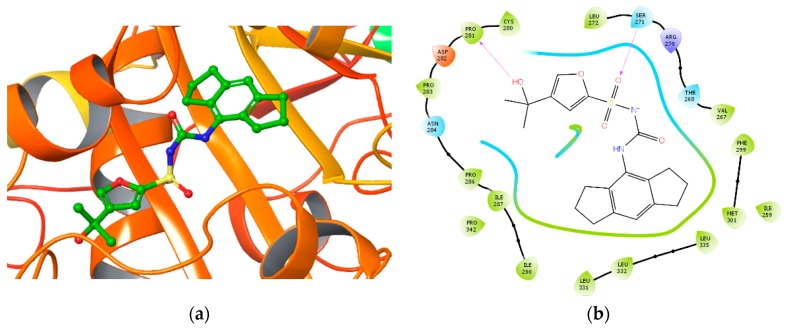
(**a**) The MCC950 pose from docking in XP precision on a Walker B centered grid; (**b**) Ligand interaction diagram of MCC950 from docking in XP precision on a Walker B centered region. Purple arrows refer to H-bond interactions, green lines around molecule underline non-polar regions whereas the blue ones point polar residues.

In particular, the following interactions were retrieved: a hydrogen bond between tertiary alcohol of MCC950 and Pro281 and Asn284, and a hydrogen bond between the sulfonyl oxygen of MCC950 and Ser271. The complex was also stabilized by hydrophobic interactions with residues Pro286, Ile287, Leu331, Leu332, Leu335, and Pro342. The best docking result of NLRP3 binding MCC950 (docking score of −8.850 Kcal/mol) is illustrated in Figure 4a, while Figure 4b shows the most significant interactions.

**Figure 5:**
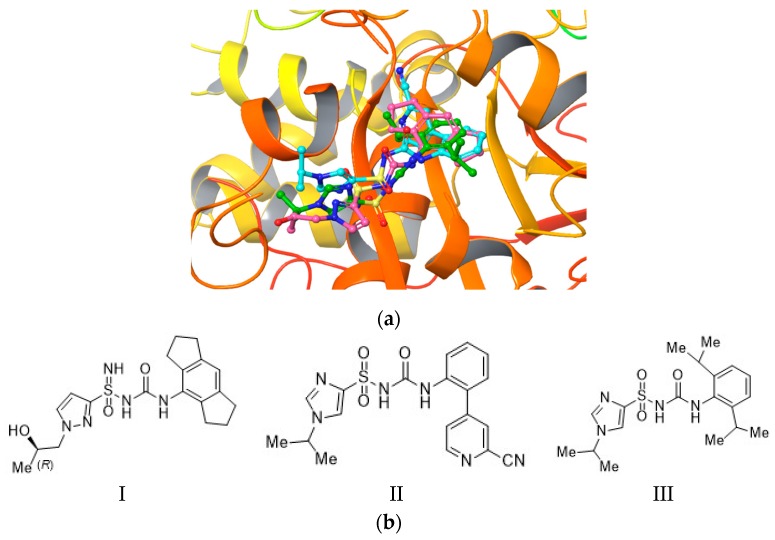
(**a**) Binding pose of compounds **I**, **II**, and **III** docked into the centered Walker B region; (**b**) compounds **I**, **II**, and **III** 2D structure.

It is noteworthy that the hydrogen bonds with Pro281 and Asn284 were also found in the previous XP docking, even though the grid included a region crossing both the Walker A and B. This interaction and preference for the Walker B domain represents indeed an interesting finding in agreement with evidences reported in the literature [4,5]. In fact, the tertiary alcohol moiety seems to be a chemical feature of several most potent MCC950 analogues, with NLRP3 inhibitory activity (IC_50_) ≤ 8 nM [2,17,18], thus indicating a critical role of this functional group in the binding recognition process. These results prompted us to investigate the binding mode of three MCC950 analogues (IC_50_ < 1 µM as reported in the patent) [19,20] on our homology model centered on the Walker B, thus XP docking was performed. We decided to use these three compounds in our docking studies, although less potent than MCC950 (8 nM), because they slightly differ from the known inhibitor. The idea was to investigate if different chemical features would still bind to the same hot spots. Figure 5 depicts the docking experiment of compounds **I**, **II**, and **III**—MCC950, sulfoximine, and sulfonylurea derivatives, respectively [19,20].

**Figure 6:**
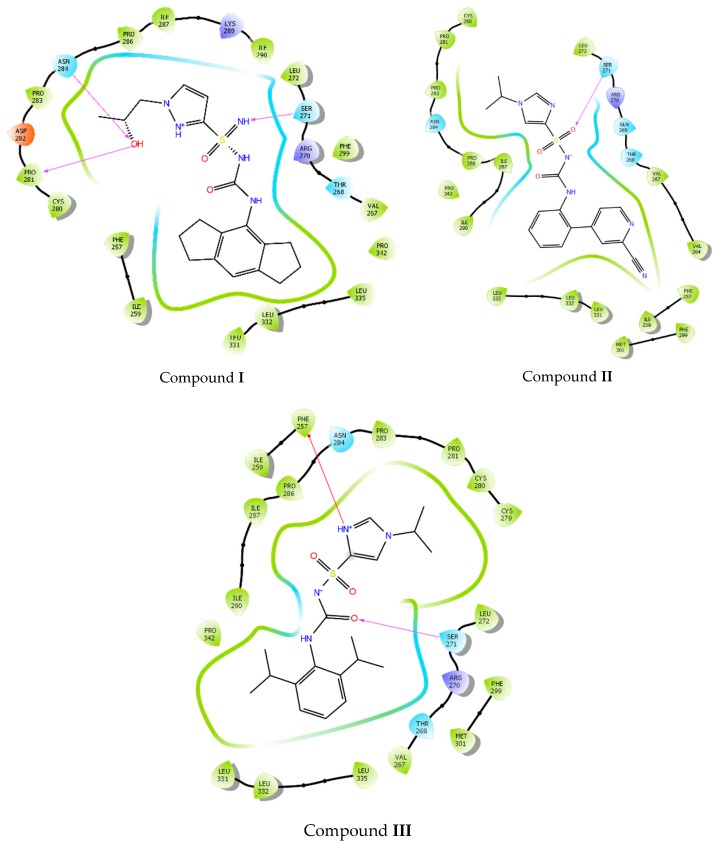
Ligand interaction diagram of compounds **I**, **II**, and **III** docked on the centered Walker B region. Purple arrows refer to H-bond interactions, green lines around molecule underline non-polar regions whereas the blue ones point polar residues.

As shown, the poses of compounds **I**, **II**, and **III** mostly converged. Pro281, Asn284, Phe257, and Ser271 residues established interactions with the ligand, in alignment with the observed previous docking results on MCC950 (Figure 6).

Moreover, starting from these docking poses, a more accurate binding affinity was evaluated with a Molecular Mechanics-Generalized Born Surface Area (MM-GBSA) method in order to estimate the binding free energy of the protein and MCC950. The MM-GBSA was calculated for the XP docking poses using Prime MM-GBSA by Schrodinger software v.2018-2 [21].

The best MM-GBSA ∆G value was −41.577 Kcal/mol. Interestingly, the binding pose identified as the best XP docking precision pose showed the lowest MM-GBSA value. From these experimental data, we could infer that Pro281 and Ser271 residues could be crucial for the stabilization of the protein–ligand complex.

In order to deepen even further our study, an induced-fit docking (IFD) (Schrödinger LLC) [22] experiment was carried out. IFD confers more flexibility to the protein side chains, allowing the ligand to adjust and optimize binding interactions within the active site. This experiment was performed by focusing the receptor grid on the Walker B motif and OPLS3e was chosen as Force field. The IFD Glide redocking protocol was set on XP precision. In particular, in the best IFD result, the hydrogen bond interaction between Ser271 residue of the protein and the MCC950 sulfonylurea carboxyl group was confirmed, while Pro281 amino acid showed a hydrogen bonding with the tertiary alcohol moiety of the inhibitor. Again, it was promising to see that Ser271 and Pro281 were recurring in the binding modes emerged from different approaches, strengthening the outcomes observed from the previous semi flexible docking experiments. The IFD results were analyzed with the MM-GBSA scoring function. The best MM-GBSA value reported was −43.187 Kcal/mol referring to the best IFD pose, involving the same interaction pattern retrieved in XP. Docking and MM-GBSA calculations were also performed on NLRC4 (the homology model template, PDBid:4KXF) to eventually speculate on the selectivity of MCC950 towards NLRP3 and/or NLRC4. In Table 1 the docking scores and MM-GBSA results are reported for comparison between the two proteins. As seen, the inhibitor seems show higher affinity for the NLRP3 protein model.

### 2.4. Molecular Dynamics

**Figure 7:**
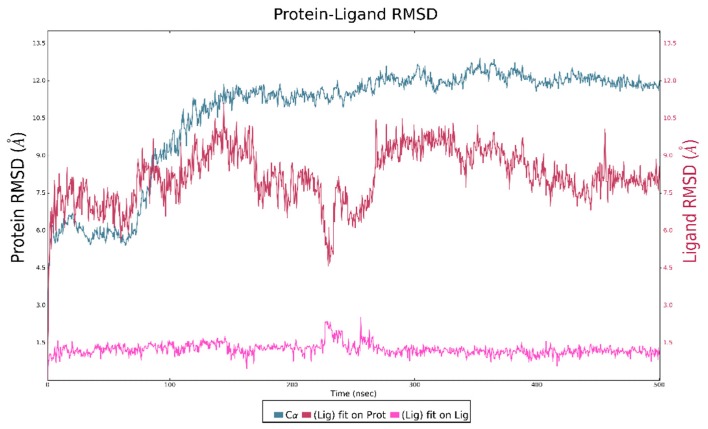
Molecular dynamics Root Mean Square Deviation (RMSD) plot.

Our investigation was further extended by performing 500 ns MD using Desmond [23], with the aim of evaluating the complex stability [24]. MD was launched starting from the best retrieved XP docking pose. The simulation complex was generated using TIP3P as the water model and an orthorhombic box. The box volume was minimized and an OPLS3e force field (OPLS3e FF) was used. Starting from the MD trajectory, Root Mean Square Deviation (RMSD) was analyzed to evaluate complex stability, as described in Figure 7.

**Figure 8:**
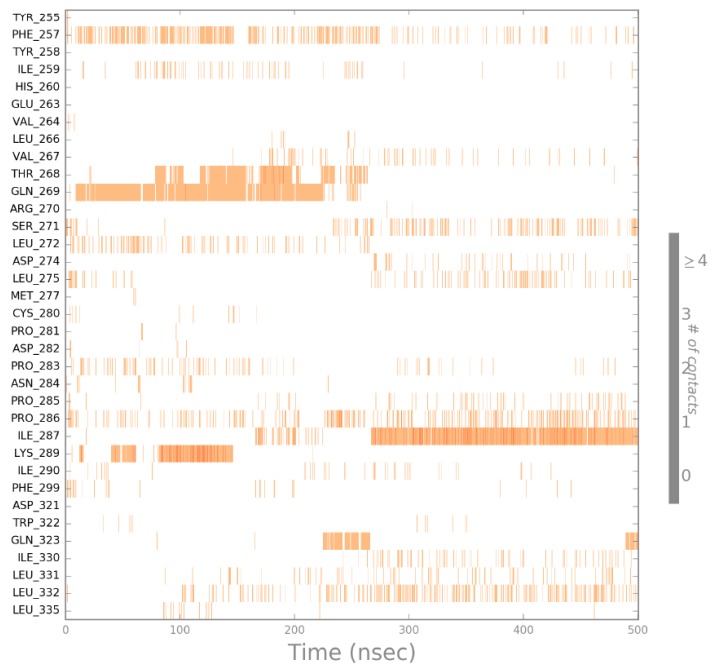
Protein–ligand contact timeline. Darker shades correspond to a higher number of contacts.

The plot analysis showed a rise in the RMSD plot around 100 ns, after which the NLRP3 protein remained stable for the whole duration of MD, with a RMSD value lower than 1. The ligand reached stability around 200 ns and until the end of the simulation. From 300 ns onwards, ligand and protein RMSD plots showed a trend with RMSD < 2 Å because of a complex stability. The protein–ligand contacts plot led us to identify the protein–ligand binding residues involved in the stabilizations of MCC950. MD trajectory showed that up to 220 ns, different residues, such as Phe257, Thr268, Gln269, Leu272, Pro283, and Lys289 seem to be important before the ligand stabilization. Then, around 200 ns, Val267, Ser271, Asp274, Leu275, Ile287, Ile330, Leu331, and Leu332 residues established contact with the ligand (Figure 8), and emerged as important hot spots for ligand stabilization.

**Figure 9:**
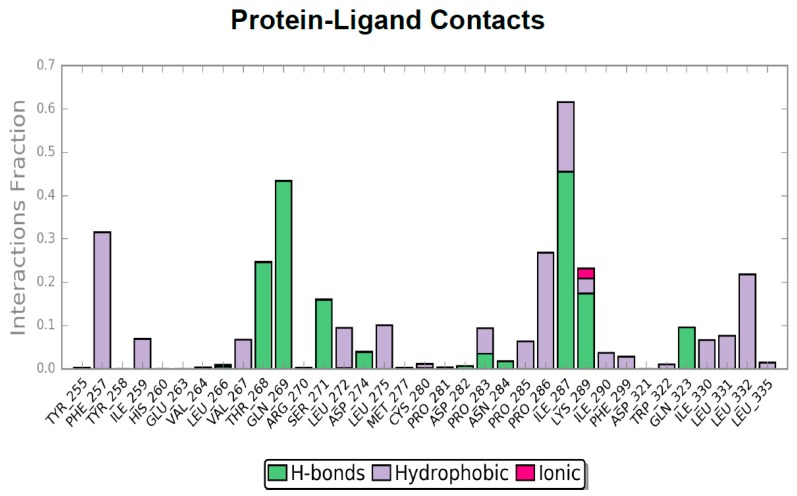
Protein interaction diagram. Green: H-bonds; Purple: Hydrophobic; Pink: Ionic.

As shown in Figure 9, Pro281 and Asn284 residues were included in the MD phase, which are reported in the docking studies to interact with the MCC950 alcohol portion, even though the number of contacts in each trajectory were low.

Gratifyingly, MD again highlighted the importance of Ser271 and Phe257 residues for the ligand stabilization, in agreement with the docking results, and showed additional residues involved in the binding of MCC950, such as Val267, Ser271, Asp274, Ile287, Ile330, Leu331, and Leu332.

## 3. Materials and Methods

### 3.1. Homology Modeling

Starting from the NLRP3 Human sequence (https://www.uniprot.org/), the homology model was built using Phyre2 (http://www.sbg.bio.ic.ac.uk/phyre2). The model obtained was refined with the Protein Preparation Wizard tool of Maestro Suite Software v. 11.6 [25] in order to optimize the protein structure improprieties, such as correct assignment of connection bonds, addition of missing hydrogens, optimization of the protonation state in a pH range 7.0 ± 2.0, and analysis of atomic clashes. Starting from the template coordinates, ATP was inserted within the catalytic site. In the given pH range, the Epik v. 4.4 [26,27] tool helped us with predicting ionization and the tautomeric state of the ATP (referring to prosthetic groups), while PROPKA was chosen to predict the protonation state of ionizable protein groups. The protein was refined using restrained minimization with OPLS3e as a Force Field. Heavy atom convergence was set with RMSD equal to 0.30 Å. The model obtained was analyzed by PROCHECK (https://servicesn.mbi.ucla.edu/PROCHECK/). When the quality of the protein structure was examined by a Ramachandran Plot (measuring the side-chains torsion angles), notably, 84.4% of the residues of our homology model fell in the most favored region.

### 3.2. Binding Site Detection

The possible binding sites on the NLRP3 homology model were searched with three binding site detectors FTMap v. 2015, SiteMap v. 2.3, and fpocket 3.0.

#### 3.2.1. FTMAP Parameters Description

Default parameters of the webserver were used to find putative binding sites [9].

#### 3.2.2. Sitemap

The Sitemap tool was used on the entire protein (all atoms in the workspace), setting at least 10 site points per receptor site and reporting up to 5 sites. Hydrophobicity parameter was set as more restrictive and a standard grid was set. The site maps identified were then cropped at 4 Å from the nearest points.

#### 3.2.3. Fpocket

Default parameters of the fpocket algorithm were used.

### 3.3. Ligand Preparation

First, the ionisation and conformation minimisation of ligand MCC950 were calculated using the Schrödinger Jaguar suite v. 10.0 [13]. In detail, 6–31G**++ basis set and B3LYP Functional were selected. The results obtained by Jaguar optimization were compared to those obtained using Schrödinger LigPrep v. 2018-2 [25]. The Force Field adopted was OPLS3e, and Epik was selected as ionization tool at pH 7.0 ± 2.0. The generation of tautomers was selected and the maximum number of conformers generated was set at 32, but only 11 were produced by the LigPrep software.

### 3.4. Molecular Docking and Induced-Fit Docking

#### 3.4.1. Molecular Docking

The docking study was performed using the Gide docking tool v. 7.9. The grid box was defined on Walker B residues (296–315). The Van der Waals radii was set at 0.8 and partial cutoff was 0.15. Ligand docking was performed in standard precision (SP) and extra precision (XP) mode with no constraints. Flexible ligand sampling, sampling nitrogen inversion, and ring conformation were used, adding bias sampling torsion penalization for amides with nonplanar conformation and adding Epik state penalties to the docking score. In order to check for binding pose convergence, the top ten poses were included within the docking output.

#### 3.4.2. Induced-Fit Docking

IFD was performed using standard protocol and the OPLS3e Force Field was chosen. The receptor box center was defined on Walker B residues (296–315) without applying any constraints. The receptor and ligand Van der Waals scaling was set at 0.5. For Prime refinement, side chains of residues within 5 Å of ligand poses were refined. The maximum of 20 poses per ligand were retained. In our study, ligands were redocked in extra precision mode (XP).

#### 3.4.3. MM-GBSA

MM-GBSA was performed with the Schrodinger Suite 2018-2. VSGB solvation model was chosen using the OPLS3e Force Field. The sampling method was set as minimized [21].

### 3.5. Molecular Dynamics

MD studies were performed with Desmond of Maestro Suite Software v. 11.6. The solvent model TIP3P [28] was used and the system was incorporated in orthorombic box. The OPLS3e Force Field was used. MD was carried for 150 ns under isothermal-isobaric (NPT) ensemble [29], using one K80 Nvidia GPU. A constant temperature of 300.0 K was maintained throughout the MD [30,31,32,33]. The system was simulated using an OPLS3e Force Field.

## 4. Conclusions

Based on our results, we suggest that the most promising key interactions could be Phe257, Val267, Thr268, Gln269, Ser271, Leu272, Asp274, Leu275, Pro281, Pro283, Asn284, Ile287, Lys289, Ile330, Leu331, and Leu332 residues, which seem to be of importance for the binding of MCC950 and derivatives, within the NLRP3 NACHT domain. Moreover, Phe257 residue was confirmed to be crucial for the stabilization of the aromatic portion of the inhibitors. Furthermore, we propose that MCC950 could bind in a region crossing Walker B and partially Walker A, thus affecting the protein conformation and inhibiting the oligomerization process. This could be in accordance with the importance of Walker A in the stabilization of MCC950 within the Walker B. Nevertheless, the binding region identified in our investigation should be further explored in view of the design of novel noncovalent NLRP3 inhibitors. Additional investigations are currently on going in our group using other simulation techniques.

## Figures and Tables

**Table 1:** Comparison between NLRP3 and NLRC4 binding evaluation.

PROTEIN	Docking Score (Kcal/mol)	MM-GBSA (Kcal/mol)
NLRP3 model	−8.85	−41.58
NLRC4 (pdbid:4KXF)	−4.01	−28.62

## Abbreviations

IFD	Induced-fit Docking
MD	Molecular dynamic simulation
NLRP3	NOD-like receptor family, pyrin domain-containing protein 3
vdW DFT	van der Waals Density Functional Theory

## References

[1] Mangan M.S.J., Olhava E.J., Roush W.R., Seidel H.M., Glick G.D., Latz E. (2018). Targeting the NLRP3 Inflammasome in Inflammatory Diseases. Nat. Rev. Drug Discov..

[2] Coll R.C., Robertson A.A.B., Chae J.J., Higgins S.C., Muñoz-Planillo R., Inserra M.C., Vetter I., Dungan L.S., Monks B.G., Stutz A. (2015). A Small-Molecule Inhibitor of the NLRP3 Inflammasome for the Treatment of Inflammatory Diseases. Nat. Med..

[3] Coll R.C., Hill J.R., Day C.J., Zamoshnikova A., Boucher D., Massey N.L., Chitty J.L., Fraser J.A., Jennings M.P., Robertson A.A.B. (2019). MCC950 Directly Targets the NLRP3 ATP-Hydrolysis Motif for Inflammasome Inhibition. Nat. Chem. Biol..

[4] Tapia-Abellán A., Angosto-Bazarra D., Martínez-Banaclocha H., De Torre-Minguela C., Cerón-Carrasco J.P., Pérez-Sánchez H., Arostegui J.I., Pelegrin P. (2019). MCC950 Closes the Active Conformation of NLRP3 to an Inactive State. Nat. Chem. Biol..

[5] Gorka O., Neuwirt E., Groß O. (2019). Walking Over The Inflammasome. Nat. Chem. Biol..

[6] Mukherjee S., Szilagyi A., Roy A., Zhang Y., Kolinski A. (2011). Genome-Wide Protein Structure Prediction. Multiscale Approaches to Protein Modeling.

[7] Moult J., Fidelis K., Kryshtafovych A., Schwede T., Tramontano A. (2018). Critical Assessment of Methods of Protein Structure Prediction (CASP)—Round XII. Proteins Struct. Funct. Bioinform..

[8] Kelley L.A., Mezulis S., Yates C.M., Wass M.N., Sternberg M.J.E. (2015). The Phyre2 Web Portal for Protein Modeling, Prediction and Analysis. Nat. Protoc..

[9] Kozakov D., Grove L.E., Hall D.R., Bohnuud T., Mottarella S.E., Luo L., Xia B., Beglov D., Vajda S. (2015). The FTMap Family of Web Servers for Determining and Characterizing Ligand-Binding Hot Spots of Proteins. Nat. Protoc..

[10] Halgren T.A. (2009). Identifying and Characterizing Binding Sites and Assessing Druggability. J. Chem. Inf. Model..

[11] Rooklin D., Wang C., Katigbak J., Arora P.S., Zhang Y. (2015). Alphaspace: Fragment-Centric Topographical Mapping to Target Protein-Protein Interaction Interfaces. J. Chem. Inf. Model..

[12] Le Guilloux V., Schmidtke P., Tuffery P. (2009). Fpocket: An Open Source Platform for Ligand Pocket Detection. BMC Bioinform..

[13] Bochevarov A.D., Harder E., Hughes T.F., Greenwood J.R., Braden D.A., Philipp D.M., Rinaldo D., Halls M.D., Zhang J., Friesner R.A. (2013). Jaguar: A High-Performance Quantum Chemistry Software Program with Strengths in Life and Materials Sciences. Int. J. Quantum Chem..

[14] Friesner R.A., Banks J.L., Murphy R.B., Halgren T.A., Klicic J.J., Mainz D.T., Repasky M.P., Knoll E.H., Shelley M., Perry J.K. (2004). Glide:  A New Approach for Rapid, Accurate Docking and Scoring. 1. Method and Assessment of Docking Accuracy. J. Med. Chem..

[15] Friesner R.A., Murphy R.B., Repasky M.P., Frye L.L., Greenwood J.R., Halgren T.A., Sanschagrin P.C., Mainz D.T. (2006). Extra Precision Glide:  Docking and Scoring Incorporating A Model of Hydrophobic Enclosure for Protein−Ligand Complexes. J. Med. Chem..

[16] Halgren T.A., Murphy R.B., Friesner R.A., Beard H.S., Frye L.L., Pollard W.T., Banks J.L. (2004). Glide:  A New Approach for Rapid, Accurate Docking and Scoring. 2. Enrichment Factors in Database Screening. J. Med. Chem..

[17] Click G., Ghosh S., Roush W.R. (2017). Compounds and Compositions for Treating Conditions Associated with NLRP Activity. Patent.

[18] Click G., Roush W., Venkatraman S., Shen D.-M., Ghosh S., Katz J., Seidel H.M., Franchi L., Winkler D.G., Opipari J.R.A.W. (2019). Compounds and Compositions for Treating Conditions Associated with NLRP Activity. Patent.

[19] Cooper M., Miller D., Macleod A., Thom S., St-Gallay S., Shannon J. (2019). Sulfonylureas and Sulfonylthioureas as NLRP3 Inhibitors. Patent.

[20] Miller D., Thom S., St-Gallay S., Shannon J., Leeson P. (2019). Novel Compounds Nouveaux Composés. Patent.

[21] Li J., Abel R., Zhu K., Cao Y., Zhao S., Friesner R.A. (2011). The VSGB 2.0 Model: A Next Generation Energy Model for High Resolution Protein Structure Modeling. Proteins Struct. Funct. Bioinf..

[22] Sherman W., Day T., Jacobson M.P., Friesner R.A., Farid R. (2006). Novel Procedure for Modeling Ligand/Receptor Induced Fit Effects. J. Med. Chem..

[23] Bowers K.J., Chow E., Xu H., Dror R.O., Eastwood M.P., Gregersen B.A., Klepeis J.L., Kolossvary I., Moraes M.A., Sacerdoti F.D. (2006). Scalable Algorithms for Molecular Dynamics Simulations on Commodity Clusters. Proceedings of the 2006 ACM/IEEE SC|06 Conference (SC’06).

[24] Perricone U., Gulotta M.R., Lombino J., Parrino B., Cascioferro S., Diana P., Cirrincione G., Padova A. (2018). An Overview of Recent Molecular Dynamics Applications as Medicinal Chemistry Tools for the Undruggable Site Challenge. Medchemcomm.

[25] Sastry G.M., Adzhigirey M., Day T., Annabhimoju R., Sherman W. (2013). Protein and Ligand Preparation: Parameters, Protocols, and Influence on Virtual Screening Enrichments. J. Comput. Aided Mol. Des..

[26] Shelley J.C., Cholleti A., Frye L.L., Greenwood J.R., Timlin M.R., Uchimaya M. (2007). Epik: A Software Program for Pk(A) Prediction And Protonation State Generation for Drug-Like Molecules. J. Comput. Aided Mol. Des..

[27] Greenwood J.R., Calkins D., Sullivan A.P., Shelley J.C. (2010). Towards the Comprehensive, Rapid, and Accurate Prediction of the Favorable Tautomeric States of Drug-like Molecules in Aqueous Solution. J. Comput. Aided Mol. Des..

[28] Izadi S., Onufriev A.V. (2016). Accuracy limit of rigid 3-point water models. J. Chem. Phys..

[29] Cheng A., Merz K.M. (1996). Application of the Nosé-Hoover chain algorithm to the study of protein dynamics. J. Phys. Chem..

[30] Fogolari F., Corazza A., Toppo S., Tosatto S.C.E., Viglino P., Ursini F., Esposito G. (2012). Studying interactions by molecular dynamics simulations at high concentration. J. Biomed. Biotechnol..

[31] Wang J., Olsson S., Wehmeyer C., Pérez A., Charron N.E., De Fabritiis G., Noé F., Clementi C. (2019). Machine Learning of Coarse-Grained Molecular Dynamics Force Fields. ACS Cent. Sci..

[32] Buchko G.W., Pulavarti S.V.S.R.K., Ovchinnikov V., Shaw E.A., Rettie S.A., Myler P.J., Karplus M., Szyperski T., Baker D., Bahl C.D. (2018). Cytosolic expression, solution structures, and molecular dynamics simulation of genetically encodable disulfide-rich de novo designed peptides. Protein Sci..

[33] Dalle Vedove A., Falchi F., Donini S., Dobric A., Germain S., Di Martino G.P., Prosdocimi T., Vettraino C., Torretta A., Cavalli A. (2019). Structure-Based Virtual Screening Allows the Identification of Efficient Modulators of E-Cadherin-Mediated Cell–Cell Adhesion. Int. J. Mol. Sci..

